# Molecular Mechanisms of Experimental Salt-Sensitive Hypertension

**DOI:** 10.1161/JAHA.112.002121

**Published:** 2012-06-22

**Authors:** Bina Joe, Joseph I. Shapiro

**Affiliations:** Center for Hypertension and Personalized Medicine, University of Toledo College of Medicine and Life Sciences Toledo, OH (B.J., J.I.S.); Department of Physiology/Pharmacology, University of Toledo College of Medicine and Life Sciences Toledo, OH (B.J., J.I.S.); Department of Medicine, University of Toledo College of Medicine and Life Sciences Toledo, OH (J.I.S.)

**Keywords:** cardiomyopathy, genes, genetic hypertension, hypertension, hypertension (high blood pressure)

## Introduction

Hypertension has been defined operationally as the level of blood pressure (BP) at which the benefits of treatment exceed the risks.^1^ It also has been defined in a more concrete way by the Joint National Committee on Hypertension (JNC-7) as, in adults, a systolic BP ≥140 mm Hg or a diastolic BP ≥90 mm Hg.^2^ Hypertension has been shown to be a risk factor for a variety of morbidities, especially stroke, myocardial infarction, and the development of congestive heart failure, as well as overall death.^3^ The prevalence of hypertension seems to increase in populations as they age, a subject that we will discuss further below. Treatment of hypertension has been shown to be extremely effective in reducing cardiovascular morbidity and mortality rates.^4^

A very strong relationship exists between dietary salt and the risk of developing hypertension. This has been observed in several clinical reports, perhaps most notably in a 1960 report by Louis Dahl, who described the possible role of salt intake in the development of hypertension.^5^ Of course, Dahl went on to develop a critical animal model for the study of salt-dependent hypertension that we will discuss further. One of the most compelling collections of data supporting the relationship between salt and hypertension was presented in the Intersalt study.^6^ Intersalt investigators, looking at populations with a wide range of sodium intake, found a correlation of salt intake with BP. Interestingly, the correlation between BP and sodium intake was dependent on inclusion of centers with very low salt intake values, values that frankly might not be relevant for most populations. Nevertheless, the correlation between sodium intake and the age-dependent increase in BP was considerably more robust and was maintained even when the extremely low-sodium-intake sites were excluded from analysis.^7,8^

Dietary sodium intake has changed dramatically during the time that humans have lived. It is believed to have been very low when humans survived primarily as hunter-gatherers and to have increased to a peak in the period of time immediately before the availability of refrigeration, when salting was the primary food preservation strategy. Advocates for lowering dietary sodium can draw some solace from the knowledge that dietary sodium, though still very high in the developed world, has reduced considerably since the availability of refrigeration. It is interesting that some debate still continues on the merits of reducing dietary sodium. Some investigators even have questioned whether there might be some harm inherent in lowering dietary sodium from current levels,^9^ but we think it is fair to say that the vast majority of workers in the field project substantial public health and individual health benefits from reducing dietary sodium intake.^10^

## Guytonian Framework

**Figure 1.**
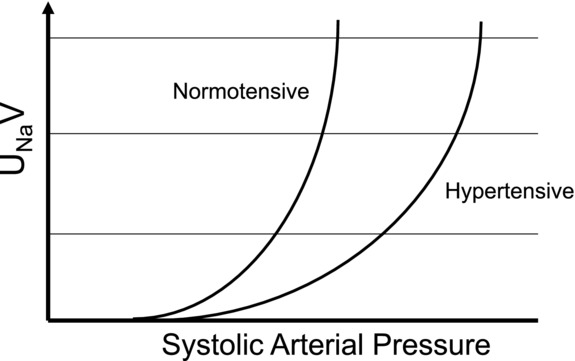
Schematic showing renal function curves from normotensive subject and hypertensive subject relating urinary sodium excretion (U_Na_V) to systolic arterial pressure for each.

The most obvious connection between sodium intake and health is manifested by the relationship between sodium intake and BP. As discussed before, although there is clearly a relationship between salt intake and BP across very wide ranges of salt intake, this is not easily seen across smaller ranges. In contrast, the relationship between changes in BP with age and dietary sodium intake is quite robust. In fact, we will discuss this latter phenomenon in some detail later in this review. However, focusing on the former relationship, the work of Arthur Guyton must be considered for a complete understanding. In the early 1960s, engineering concepts had made their way into our understanding of hemodynamics and, by extension, BP regulation. Arthur Guyton, working with an engineer, Thomas Coleman, made a minor but important twist to the accepted models of BP control, putting in terms for renal salt (actually, initially water) excretion and dietary salt (again, initially water) intake. To the surprise of the scientific community, the relationship between BP and renal salt excretion, referred to as a *renal function curve* (see Figure 1), proved to be, along with dietary sodium intake, a key controller of long-term BP in these computer simulations.^11,12^ This concept has been tested in both animal models and clinical transplantation studies, and though many would not consider it a complete explanation, it is fair to say that the renal function curve and dietary sodium excretion are considered important regulators of BP.^13^ It almost goes without saying that in patients who are relatively resistant to salt, their renal function curve is described by a low set point and a very steep relationship, whereas those who are salt sensitive can be described by a shifted set point and a less steep relationship (Figure 1).

It is only fair to point out that we have presented a fairly catholic rendition of Guyton's concept. There are many workers in hypertension who have presented considerable data disputing the integral nature of this concept for both clinical and experimental hypertension. Although the authors of the present review are not profoundly devout in our acceptance of Guyton's concept from all corners, we would argue that it certainly does apply to a considerable subset of clinical and experimental hypertension. In fact, within this framework, one might interpret the Intersalt study as demonstrating that high salt intake predictably shifts the average renal function curve of a population with time to one favoring salt-sensitive hypertension.^7^

## Genetics of Experimental Hypertension

The most studied rat model of salt sensitivity and hypertension originally was developed by Lewis Dahl in the 1960s at the Brookhaven National Laboratory (Upton, NY).^14,15^ These rats were developed from Sprague Dawley rats that were selected and bred on the basis of their BP after being fed a very high-salt (8% NaCl) diet and are called the Dahl salt-sensitive and Dahl salt-resistant rats.^14^ Within 3 generations of selection, the salt-sensitive and salt-resistant strains were clearly different, which suggested, for the first time, that salt sensitivity was an inherited trait. All of Dahl's reports on the spectacular divergence in BP between these strains in response to dietary salt were based on studies on these outbred strains.

To delineate the inherited factors contributing to salt sensitivity and the development of hypertension, inbred strains were developed from these outbred stocks in the United States by Professor John Paul Rapp at the Medical College of Ohio in Toledo (now the University of Toledo College of Medicine and Life Sciences) and in Japan by Iwai and Heine.^16^ The inbred strains developed in the United States are designated as SS/Jr (salt-sensitive; S) and SR/Jr (salt-resistant; R) rats.^17^ These are commercially available through Harlan Sprague Dawley, but the inbred SS/JrHsd rats commercially available from Harlan Sprague Dawley were genetically contaminated in the 1990s, with the contamination apparently rectified thereafter. This strain from Harlan Sprague Dawley (SS/JrHsdMcw or SS/Mcw) was used in most studies from the Medical College of Wisconsin. However, at the genomic level, the data available at the rat genome database (SNPlotyper at http://www.rgd.mcw.edu) indicate that the original SS/Jr is 0.2% polymorphic compared with SS/JrHsd and 2.46% polymorphic compared with SS/JrHsdMcw.

Using the most definitive colonies maintained at our institution, we investigated the role of sodium potassium adenosine triphosphatase (Na/K-ATPase) signaling in renal sodium excretion and BP regulation.^18^ High salt in the diet significantly reduced Na/K-ATPase activity in the R rats, which was accompanied by notable phosphorylation of c-Src and extracellular signal–related kinases 1 and 2 and reduced sodium–hydrogen exchanger isoform 3 (NHE3) activity. These responses were either not seen or much less effective in the S rats.^18^ These differences also were observed within isolated primary proximal tubule cell cultures, in which ouabain induced Na/K-ATPase/c-Src signaling and redistribution of the Na/K-ATPase and NHE3 in the Dahl R rats but not in the Dahl S rats.^18^ These functional contrasts are not due to inherent structural variations of *Atp1a1*, the gene coding for the α subunit of Na/K-ATPase, because Mokry and Cuppen demonstrated that between the S and R rats there are no coding sequence variations.^19^ Collectively, our data suggest that impairment of Na/K-ATPase signaling and consequent regulation of Na/K-ATPase and NHE3 in renal proximal tubules may contribute to salt-induced hypertension in the Dahl S rat. Given that these pathophysiological differences are observed between the 2 strains S and R that were originally selected for inherited factors controlling BP, it is highly likely that some of the overriding factors governing the differences in Na/K-ATPase are due to yet-unidentified inherited factors influencing hypertension.

Hypertension is known to be inherited in families, which means that there is definitive evidence for the presence of genetic elements on our genomes that predispose some of us to develop high BP at some point in our lifetimes. Investigation into this overwhelming evidence for genetic factors to control the extent of our BP has led to some important gene discoveries for monogenic forms of hypertension, which are caused essentially by functional defects of single gene products.^20–26^ Essential hypertension, which represents >90% of all forms of hypertension, is, however, a polygenic trait (ie, caused by many genes). Discovering the genetic basis of essential hypertension has remained a daunting task. This challenge is nevertheless important to undertake because it leads to a better understanding of the fundamental “blueprint,” or genetic architecture, of the causative pathophysiological mechanisms that result from concerted action of alterations in the identified genes.

## Genome-Wide Association Studies, “Missing Heritability,” and Epigenetics of Hypertension

Driven by the advances in sequencing technology, in recent years, large-scale genome-wide association strategies have been applied to uncover the identities of genes associated with hypertension. Although several of these genome-wide association studies (GWAS) have identified a number of genes as associated with hypertension,^27–33^ collectively, these studies account for <1% of the 25% to 30% estimated contribution of genes to the trait of BP. One of the reasons for this disappointing situation is that by nature of their design, GWAS are intended to detect the effects of common gene variants on BP and have limited ability to detect rare variants that affect BP.^34^ A second, more complicated explanation for “missing heritability” is that the genetics of BP control share a feature common to many complex traits: They encompass not only the effects of many genes, but also gene–gene and gene–environment interactions. In a recent article,^35^ Zuk and colleagues propose the following intriguing thought on this topic:

The proportion of heritability explained by a set of variants is the ratio of (*i*) the heritability due to these variants (numerator), estimated directly from their observed effects, to (*ii*) the total heritability (denominator), inferred indirectly from population data. The prevailing view has been that the explanation for missing heritability lies in the numerator—that is, in as-yet undiscovered variants. Although many variants surely remain to be found, a substantial portion of missing heritability could arise from overestimation of the denominator, creating “phantom heritability.” Specifically, (*i*) estimates of total heritability implicitly assume the trait involves no genetic interactions (epistasis) among loci; (*ii*) this assumption is not justified, because models with interactions are also consistent with observable data; and (*iii*) under such models, the total heritability may be much smaller and thus the proportion of heritability explained much larger.

In addition to rare, yet-undetected variants and epistasis as factors contributing to the missing heritability, an evolving trend in hypertension research is to address the hypothesis that epigenetic factors are causal but not consequential as a result of high BP. *Epigenetics* refers to mechanisms for environment–gene interactions, such as methylation of DNA and modification of histones, which *do not* alter the underlying base sequence of the gene. Therefore, it is clear that strategies aimed at detecting genomic variants are highly likely to miss the detection of epigenetic factors. Studies aimed at identification of such epigenetic factors for hypertension are beginning to accumulate associations of epigenetic events involving nephron development, expression of renin–angiotensin system genes, and blood vessel remodeling.^36–39^ However, to address whether epigenetics contributes to the missing heritability of hypertension, it is imperative that study designs be focused on differentiating the “causal” epigenetic factors for hypertension from the “consequential” epigenetic factors associated with hypertension. A recent National Heart, Lung, and Blood Institute working group report^40^ further emphasized this aspect of epigenetic research in hypertension and concluded that, though challenging, integration from both model organism research and human epigenetics might be required to pinpoint a causal relationship (and not mere association) of epigenetics with hypertension.

In any case, one way of interpreting the genetics of BP control is to view an individual's BP as the sum effect of the gene products of all the independent and interactive susceptibility genes that drive BP to be elevated plus the gene effects of all the genes that confer resistance to the development of elevated BP. Pinpointing such susceptibility-conferring genetic factors that contribute to increase the BP of an individual is obviously difficult in the context of other genes that confer strong resistance to the development of hypertension. In other words, genomic background plays a very important role in defining the ability to detect inherited elements that control BP. In this context, because the S rat was selectively bred for elevated BP in response to salt, the S rat genome could be viewed as a relatively “enriched” pool of inherited factors that function either independently or interdependently to raise BP above that of the R rat. To identify these inherited factors, the genome of the S rat is compared with that of other inbred rat strains with relatively normal BP by 2 classical methods: linkage analysis and substitution mapping with congenic strains.^17,41,42^ In the original stock of S rats available at the University of Toledo, 8 different linkage analyses resulted in the identification of at least 16 different genomic segments harboring genetic determinants of BP.^41–43^ Other laboratories have similarly mapped additional genomic segments by comparing the S rat genome with other normotensive strains.^44–62^ The details of all these studies are cataloged elsewhere.^17,41,42,44,52^ For the purpose of the present review, the most advanced of these reports in which mapping has been achieved in the original stock of S/Jr rats, as well as the translational relevance of the studies to human hypertension, are presented below. The prioritized genes are listed in the Table 1 along with their locations on the rat genome and the corresponding homologous regions on the human genome.

**Table 1.** Candidate Blood Pressure–Controlling Genomic Loci Prioritized by High-Resolution Substitution Mapping Studies in the Original Stock of S/Jr Rats

Rat Locus Prioritized by High-Resolution Substitution Mapping of the S/Jr Rat	Gene Symbol	Affected Molecular Mechanism	Genomic Size of the Mapped Location	Mapped Location on the Rat Genome (Rat Chromosome Number: From Base Pairs to Base Pairs)	Homologous Human Genomic Segment (Human Chromosome Number: From Base Pairs to Base Pairs)	Genetic Association to Human Cardiovascular Disease
11β-Hydroxylase	*Cyp11b1*	Steroid biogenesis	177 kb	7: 112 800 232–112 978 080	8: 143 720 961–143 928 382	Yes^63^

A disintegrin-like metalloproteinase with thrombospondin motifs, 16	*Adamts16*	Unknown	804.6 kb	1: 30 876 304–31 680 901 (*17: 2 374 083–3 178 680)	5: 4 859 244–5 888 257	Yes^64^

Rififylin	*Rffl/Carp-2*	Endocytic recycling in cardiomyocytes and proximal tubules	42.5 kb	10: 71 028 112–71 070 581	17: 33 342 355–33 397 897	Yes^65^

Nuclear receptor subfamily 2, group F, family 2	*Nr2f2*	Transcriptional networks with other transcription factors	7.4 Mb†	1: 132 162 132–139 471 537	15: 92 108 680–99 502 957	Yes^66^

All mapped locations on the human genome were obtained by blast searching the human genome assembly with rat genome sequences at http://www.ensembl.org.

* This region is erroneously mapped to rat chromosome 17 in multiple online rat genome databases. The correct location on rat chromosome 1 is obtained from the Celera rat genome assembly. For details, please see Joe et al.^64^

† This is not considered very high-resolution mapping but is mentioned here because of the parallel observation that *Nr2f2* is a very highly prioritized gene in a human GWAS.

Mutations in the gene coding for 11β-hydroxylase, *Cyp11b1*, were the first to be linked to the development of hypertension in the Dahl S rat. *Cyp11b1* is located on rat chromosome 7 and human chromosome 8 (Table 1). Dahl S rat adrenals produce more 18-hydroxy-11-deoxycorticosterone (18-OH-DOC) than do R rats.^67,68^ This differential steroidogenic pattern segregates in a Mendelian fashion and cosegregates with BP. Enzymatic studies^69^ showed that genetic variants in *Cyp11b1* caused the observed alterations in steroidogenesis. *Cyp11b1* catalyzes both the 18- and 11β-hydroxylation of 11-deoxycorticosterone to form 18-OH-DOC and corticosterone, respectively.^70^ Five single-nucleotide polymorphic variants are reported between S and R rats that result in substitutions of amino acids 127, 351, 381, 384, and 443.^69,73^ Of these, substitutions at 381, 384, and 443 but not those at 127 and 351 are further demonstrated to alter the strain-specific steroidogenic pattern.^71^ Residues at 381 and 384 are thought to be in or near the substrate recognition site and are demonstrated to be required, and almost sufficient, for full expression of the difference in 18-OH-DOC production characteristic of S and R rats.^72^ Not surprisingly, linkage analysis between S and R rats pointed to a large genomic segment on chromosome 7 encompassing *Cyp11b1* as a candidate genetic determinant of BP.^75^ Average survival on a high-salt (4% NaCl) diet was markedly increased in the *Cyp11b1* congenic strain as compared with S, and the difference in survival was accounted for by the differences in BP.^73^ Multiple iterations of congenic substrains were further constructed by introgressing shorter introgressed segments with R alleles into the S strain, and results from the BP measurements of these congenic strains further confirmed the candidacy of *Cyp11b1*.^67,74^ Final proof-of-principle that *Cyp11b1* indeed is the most likely genetic determinant of BP that maps on rat chromosome 7 came from a high-resolution substitution mapping study with a 177-kb congenic segment containing R alleles of *Cyp11b1* on the S genome.^75^ The plausible mechanism of action of the gene product of *Cyp11b1,* steroid 18-OH-DOC, is that it is mildly hypertensinogenic, and it is produced in the zona fasciculata of the adrenal gland under the control of adrenocorticotropic hormone.^17^ On a low-salt diet, aldosterone dominates the total mineralocorticoid status of the rat. High dietary salt suppresses aldosterone but not 18-OH-DOC production. Thus, 18-OH-DOC contributes significantly to the net mineralocorticoid status of the rat. The genetic variants of *Cyp11b1* facilitate the increased plasma 18-OH-DOC concentration in S compared with R rats,^70,71,76^ and therefore, the hypertension of S rats can be expected to have a mineralocorticoid-induced component when they consume a high-salt diet.

A broad genomic region on rat chromosome 1 was identified as a locus for BP control through linkage analysis between S rats and the relatively normotensive Lewis (LEW) rat.^77^ Since then, a series of congenic strains was developed by introgressing LEW genomic segments onto the genome of the S rat.^78,79^ The BP measurements of these strains confirmed that there are at least 3 independent loci within the genomic segment identified by linkage analysis.^79^ One of these loci was further resolved to <793.5 kb containing 2 protein-coding genes.^64,80^ Coding sequence variants were detected between only one of these 2 protein-coding genes: *ADAMTS16*.^64^ *ADAMTS16* in humans is located on human chromosome 5 (Table 1 *ADAMTS16* is a member of the A disintegrin-like metalloproteinase with thrombospondin motifs (ADAMTS) gene family with no known function.^81^ There are 21 members of the ADAMTS family of metalloproteinases, and more are being found. They are recognized to be important for regulation of the turnover of extracellular matrix proteins in several tissues.^82^ Altered regulation of ADAMTS proteins has been implicated in diseases such as arthritis, cancer, and atherosclerosis.^82^ Although functional studies to delineate the mechanism of action of the natural variants of *ADAMTS16* in causing hypertension are ongoing, it is important to note that critical evidence is available to demonstrate the direct linkage and association of variants of *ADAMTS16* with human essential hypertension.^64^ Linkage and association studies with samples from the Quebec Family Study and association studies with samples from GenNet, a “genetic determinants of high BP” network, confirmed that variants of human *ADAMTS16* are both linked and associated with hypertension in humans.^64^ This mapping study therefore serves as an example of a genetic analysis in the S rat genome that has led to the prioritization of *Adamts16* as a novel candidate genetic determinant of human essential hypertension.

Nuclear receptor subfamily 2, group F, member 2 *Nr2f2*, also known as chicken ovalbumin upstream promoter transcription factor, *Coup-TfII*, was first identified as a positional candidate BP locus within a second region on rat chromosome 1 ^66^ homologous to human chromosome 15q26 (Table 1). Nr2f2 is a member of the steroid/thyroid nuclear receptor family of ligand-dependent transcription factors that was recently reported to be a critical player in controlling mesenchymal differentiation. Interestingly, 5 additional independent studies in rats and 4 independent studies in humans have reported genetic linkage for BP control by regions on the human genome that are homologous to the rat segment containing *Nr2f2*.^83–86^ *Nr2f2* was identified as a differentially expressed positional candidate gene between S and congenic strains with introgressed LEW alleles on the S genome.^66^ By the integration of results from whole-genome transcriptional profiling and custom candidate gene profiling, a complex network of transcriptional control was recognized as being initiated by the positional candidate *Nr2f2*.^66^ Final proof that this network is the underlying mechanism would require additional mapping studies in rats. Recently, Nr2f2 was found to be a factor binding to the hormone response element within the renin promoter. Knockdown of *Nr2f2* augmented the induction of renin expression by retinoic acid.^87^ In addition, 2 separate lines of evidence point to the translational significance of *Nr2f2* as a candidate gene for human hypertension. First, the genomic segment on human chromosome 15q26 containing *NR_2_F_2_* was 1 of 6 moderately associated regions for hypertension identified by the Wellcome Trust Case Control Consortium^88^ Second, a reanalysis of the Wellcome Trust Case Control Consortium data by haplotypic analysis points to a CAA haplotype within the human *NR_2_F_2_* gene as the only region that is associated with very high significance to hypertension.^89^

Several mapping studies point to candidate genes on human chromosome 17 as being plausible for BP control in humans.^85,90,91–94^ Human chromosome 17 is homologous to rat chromosome 10, which has been found to harbor several rat BP loci.^41,42,90^ Similar to mapping on chromosome 1, mapping in S and LEW rats has progressed to resolutions of a few kilobases and has revealed a complex pattern of multiple, opposing BP determinants that are closely linked.^65,77,95–97^ The best resolution of mapping is within 42.5 kb containing a single gene, *Rffl,* which encodes rififylin.^65^ The rat chromosome 10 genomic segment harboring *Rffl* in rats is homologous to human chromosome 17 (Table 1 Interestingly, a large meta-analysis of 3 GWAS in 13,685 individuals of European ancestry from the Framingham Heart Study, the Rotterdam Study, and the Cardiovascular Health Study, as part of the QTGEN consortium, found an association of short-QT intervals with multiple minor alleles on human chromosome 17 in the vicinity of the human *RFFL* gene.^98–101^ (QT is a measure of the time between the start of the Q wave and the end of the T wave in the heart's electrical cycle.) We confirmed that the rat genomic segment containing *Rffl* is similarly linked to QT intervals^65^ in rats and therefore serves as a functional validation of these GWAS. Our observation in rats that early changes in the QT interval contribute to the development of hypertension suggests that these individuals could be at risk for developing hypertension.

Unlike *Cyp11b1* and *Adamts16*, there are no coding sequence allelic variations of *Rffl* between S and LEW. However, rififylin was expressed higher in the hearts of congenic strains with LEW alleles of *Rffl* compared with S.^65^ This suggests that a variation within the promoter for *Rffl* or within a regulatory RNA molecule that controls the expression of *Rffl* could be the underlying genetic determinant of BP. Nevertheless, overexpression of rififylin caused delayed endosomal recycling within single cardiomyocytes^65^ and proximal tubules (unpublished observation) and was associated with higher accumulation of polyubiquitinated proteins.^65^ Overall, the mechanism of altered endosomal recycling accounting for altered cellular homeostasis could be prioritized as a potential novel mechanism facilitated by *Rffl* that accounts for a change in BP.

The results of the genetic studies detailed above clearly demonstrate that a permissive genomic background such as that of the S rat has been instrumental to not only systematically dissect and understand the genetics of hypertension through high-resolution mapping studies but has also led to the identification of novel molecular mechanisms for further consideration in human studies.

There is, however, one problem with mapping with the congenic approach alone. Even with some of the best resolutions, because of its dependency on naturally occurring recombinations, mapping by substitution of genomes between strains lacks the power to precisely define prioritized variants as the sole genetic determinants without any flanking genomic polymorphisms. To circumvent this problem, newer methods of functional validation are becoming available in the rat.^102–104^ These include the targeted genome-editing strategies that use zinc-finger nucleases^103,105^ and transcription activator–like effector nucleases.^106^ Feng et al^107^ recently have used the zinc-finger nucleases approach to further the substitution mapping of a 16-Mb region on rat chromosome 13 and to validate a genetic mutation within the promoter region of the S allele of a subunit of NAD(P)H oxidase, *p67(phox),* which caused a higher promoter activity than that of the salt-resistant Brown Norway rat. S rats with targeted disruption of *p67(phox)* demonstrated a significant lowering of salt-sensitive hypertension, renal medullary oxidative stress, and injury. This study fundamentally links pro-oxidant stress caused in the kidneys to salt-sensitive hypertension via variants in the promoter region of *p67(phox)*.

Together with the aforementioned genome-editing strategies, the ability to obtain and genetically engineer embryonic stem cells of the S rat is highly likely to serve as a method to validate the genetic elements that are positionally cloned via the substitution mapping approach. Thus, high-resolution mapping projects to identify inherited factors for hypertension are well poised to move toward the goal of developing novel molecular targets as avenues for clinical management of hypertension.

## Molecular Physiological and Pathophysiological Studies

As discussed previously, genetic studies have focused our attention on several inheritable factors. Clearly, this is an extremely powerful approach to this problem. Nevertheless, more detailed biochemical studies with salt as an environmental factor will be required before these novel findings can be integrated into the current framework of knowledge about the overall etiology of salt-sensitive hypertension. Regardless of salt, when BP values are low, it is clear that the renin–angiotensin–aldosterone and sympathetic nervous systems are key for restoring homeostasis.^108^ With volume expansion, natriuretic peptides initially described by de Bold and Bencosme^109,110^ also have been proposed as important for maintenance of BP. Although several important hormonal and cytokine systems have been implicated and intensively studied by laboratories across the world,^111,112^ we will focus this review on another factor, collectively referred to as the cardiotonic steroids (CTS; also called endogenous digitalis-like substances),^113^ which are emerging as a class of hormones mechanistically linked to natriuresis. Our choice to focus on the CTS is almost entirely related to the interest of our research laboratories rather than to the magnitude of their importance in experimental salt-sensitive hypertension. We will, however, argue that the importance of these hormones has been underappreciated.^113–115^

On the basis of numerous experimental observations, de Wardener and others postulated that a humoral prohypertensive factor implicated in the pathogenesis of NaCl-sensitive hypertension is an endogenous natriuretic hormone.^116^ Because Na/K-ATPase comprises a major sodium-transporting mechanism in the kidney, and because digitalis glycosides are specific ligands of the Na/K-ATPase, it has been further postulated that a putative natriuretic hormone has digitalis-like properties.^117^ According to the “concept of natriuretic hormone,” the primary role of endogenous digitalis is to promote natriuresis via inhibition of Na/K-ATPase and sodium reabsorption in the renal proximal tubules. The increased plasma levels of digitalis-like CTS also could contribute to vasoconstriction, via inhibition of the Na,K pump coupled with activation of Na^+^/Ca^2+^ exchange in vascular smooth muscle.^118^ In 1991, Hamlyn and colleagues^119,120^ identified endogenous ouabain, a well-known CTS initially found in plants, in human plasma. This suggested that ouabain was the key CTS involved in renal salt handling. However, ouabain exhibits high affinity for the α2 and α3 isoforms of Na/K-ATPase, whereas tubular cells of the mammalian kidney express mainly the α1 isoform, which is relatively insensitive to ouabain.^121^ Although endogenous ouabain does not seem likely to be natriuretic at the concentrations seen in vivo, a body of evidence emerged indicating that brain endogenous ouabain plays an important role in the pathogenesis of NaCl-sensitive hypertension.^122^ Ouabain can be classified as a cardenolide CTS. Bufadienolides are CTS that cross-react with antibodies against digoxin but differ from cardenolides such as digoxin and ouabain in having a 6-membered lactone ring.^123^ Bufadienolides initially were noted to be secreted by the skin of amphibians (eg, *Bufo marinus*) and are known to be regulated in concert with changes in environmental salt content.^113^ It now appears certain that one of the circulating mammalian CTS is the bufadienolide marinobufagenin (MBG). MBG, at low concentrations, induces vasoconstriction in isolated human blood vessels and exhibits higher, in contrast to ouabain, affinity to the α1 isoform of the Na/K-ATPase, which is the exclusive sodium pump isoform in renotubular epithelium.^124^ Recently, Komiyama et al^125^ purified MBG and telocinobufagin, a possible MBG precursor, from human plasma and found that levels of telocinobufagin and MBG were significantly elevated in plasma of patients with end-stage renal disease. Fedorova et al^126^ demonstrated that the natriuretic response to acute NaCl loading and to intrahippocampal administration of ouabain in Dahl salt-sensitive rats was associated with increases in the circulating concentration and renal excretion of MBG, and administration of an anti-MBG antibody reduced renal sodium excretion and increased activity of the Na/K-ATPase in renal medulla. Although the greater sensitivity of the renal Na/K-ATPase to MBG as opposed to ouabain may explain some of this phenomenon, another explanation may involve CTS-induced endocytosis of the Na/K-ATPase in kidney tissues, an observation that we have reported.^127–131^ We now know that CTS also appear to induce the coordinated redistribution of the sodium proton antiporter isoform 3, which is responsible for apical sodium uptake along with the endocytosis of the basolateral Na/K-ATPase, both processes that depend on signaling through the Na/K-ATPase.^132^ These results suggest that increases in the circulating concentrations of MBG result from salt loading which, in turn, induces decreases in both basolateral and apical sodium transport in the proximal tubule.

**Figure 2.**
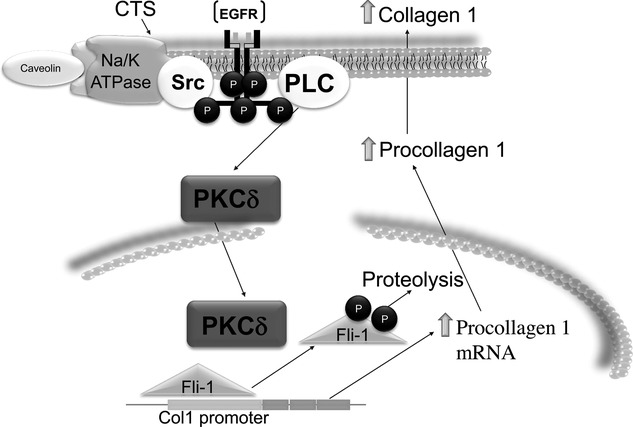
Schematic demonstrating how signaling through Na/K-ATPase might produce fibrosis. EGFR indicates epithelial growth factor receptor; PLC, phospholipase C; solid black circle labeled P, phosphate produced by phosphorylation; and PKCδ, protein kinase C isoform δ.

As mentioned previously, the classic concept for CTS signaling through the Na/K-ATPase is that the CTS inhibits the enzymatic function of the pump, producing increases in cytosolic sodium that cause a change in sodium–calcium exchange and effect increases in cytosolic calcium.^118^ However, data reported by Xie et al^133^ have cast considerable doubt on the exclusivity of this mechanism. In the late 1990s, it was noted that in neonatal cardiac myocytes, the administration of ouabain resulted in consistent increases in reactive oxygen species (ROS) detected with the fluorescence indicator, CMDCFH.^133^ Supporting the concept that these ROS were key to the signaling function of the Na/K-ATPase, it was demonstrated that the gene transcription effects of ouabain could be blocked by administration of either N-acetyl cysteine, which allows cells to detoxify ROS, or the ROS scavenger vitamin E. Additional evidence for ROS in physiological and pathological Na/K-ATPase signaling includes increased tissue and circulating protein carbonylation and the lipid oxidation byproduct malondialydehyde, as well as tissue dihydroethidium staining.^134–138^ Admittedly, the measurement of ROS in biological samples, particularly with CMDCF, is still extremely challenging and fraught with potential artifacts.^139^ Further demonstration that ROS are key in pump signaling has come in a number of settings that are beyond the scope of this discussion.^133,136,140,141^ The next step examined by our laboratories was to determine which signaling partners of the Na/K-ATPase allowed for the generation of ROS to result from the binding of CTS to the Na/K-ATPase. First, it was noted that Ras activation was a necessary step for ROS to be generated.^140^ Next, it was determined that the caveolar Na/K-ATPase normally bound Src and maintained Src in an inactive state. However, when CTS altered the Na/K-ATPase structure, Src became activated, transactivated the epidermal growth factor receptor, and thus triggered a signal cascade that resulted in ROS activation.^142–145^ In addition to the generation of ROS, the site(s) of generation and targets of which are still being worked out, activation of phospholipase C, phosphoinositide 3-kinase, and protein kinase C, as well as a number of downstream targets, has also been established.^146–150^ Still, it must be stressed that our understanding of just how ROS are produced in this signaling cascade and where they act is still incomplete. A schematic of this signaling cascade as we currently understand it is shown in Figure 2.

The possibility that CTS signaling may lead to fibrosis was also reported from our laboratories. We believe this is relevant to hypertension not only in that cardiac fibrosis is an important morbidity associated with hypertension, but because renal fibrosis would be expected to be associated with a shift in the Guytonian renal function curve favoring the development of hypertension. First, we noted that experimental renal failure produced a tremendous amount of cardiac fibrosis in both rat and mouse.^134^ Interestingly, we believe that the cardiomyopathy associated with human renal failure is also complicated by considerable fibrosis, although fibrosis, in general, does appear to develop much faster in the rodent models. Active immunization against an MBG-albumin conjugate that resulted in a high titer-specific response to MBG, as well as reduction of circulating levels of MBG by adrenalectomy, prevented the cardiac fibrosis seen with experimental renal failure, and treatment of animals with an infusion of MBG that achieved similar plasma levels of MBG as seen with experimental renal failure caused a similar degree of cardiac fibrosis. Activation of Na/K-ATPase signaling, as evidenced by increases in both Src and mitogen-activated protein kinase phosphorylation in cardiac tissue, was also seen along with the cardiac fibrosis.^134,136,137,151^ We also have observed that passive immunization is remarkably effective at preventing and even reversing evidence for Na/K-ATPase signaling and tissue fibrosis.^138^

On the basis of the in vivo results, we examined whether MBG and other CTS had effects on fibroblasts grown in culture. First, we saw that both MBG and other CTS (eg, ouabain, digoxin) caused fibroblasts grown to confluence to increase proline incorporation and collagen production, with the latter measured with Western blot. This was again coincident with evidence of Na/K-ATPase signaling in that Src and mitogen-activated protein kinase activation could be observed, as well as the effectiveness of ROS scavenging and Src inhibition in preventing increases in proline incorporation and collagen production. Increases in mRNA for collagen after exposure to MBG also were noted. Interestingly, we did not note increases in transforming growth factor-β or SMAD proteins, but antagonism of the transforming growth factor-β system with SB421542 did block MBG-induced stimulation of collagen production.^136^ On the basis of exciting work performed by the laboratory of Dr Dennis Watson in dermal fibroblasts, demonstrating that Friend leukemia integration 1 transcription factor (Fli-1) is a negative regulator of collagen synthesis,^152^ we chose to examine whether MBG signaling altered the expression of Fli-1. We noted that MBG induced decreases in Fli-1 expression in several types of fibroblasts (cardiac, renal, and dermal), and we further noted that the decreases in Fli-1 seemed to be necessary for MBG to induce increases in collagen. Further studies demonstrated that MBG appears to induce translocation of protein kinase C isoform δ from the cytosol to the nucleus in a phospholipase C–dependent manner, and that the translocation of protein kinase C isoform δ appeared to result in the phosphorylation and subsequent degradation of Fli-1.^146^ Because of these exciting results, as well as controversy as to how mineralocorticoid antagonists ameliorated cardiac fibrosis, we examined the effects of spironolactone and its major metabolite, canrenone, in a series of in vitro and in vivo studies. As anticipated, we observed that both spironolactone and canrenone could attenuate MBG-induced increases in collagen production in cardiac fibroblasts, a finding that was corroborated in vivo by marked attenuation of the cardiac fibrosis caused by experimental renal failure with spironolactone treatment. However, in vitro, we could not see a substantial effect of aldosterone on cardiac collagen production. That said, we did observe that both spironolactone and canrenone prevented MBG signaling. Moreover, both spironolactone and canrenone seemed to act as competitive inhibitors of CTS binding to the Na/K-ATPase,^153^ a finding first proposed by Finotti some 25 years ago.^154,155^ On the basis of these findings, it appears that CTS signaling may be a fertile area for therapeutic drug development.

As mentioned previously, the effects of MBG (and other CTS) are not specific for cardiac fibroblasts. In fact, renal fibroblasts have a very similar response to that of cardiac fibroblasts. This suggested to us that MBG potentially might be involved in renal fibrosis. Moving back to the in vivo model of MBG infusion, we observed that such infusion was associated with increases in renal collagen content. We also observed that Snail, a transcription factor known to be involved in epithelial–mesenchymal transformation, appeared to be upregulated with MBG infusion. LLC-PK1 cells can be induced to undergo epithelial–mesenchymal transformation by exposure to MBG in a dose- and time-dependent fashion.^156^ Most recently, we have examined whether CTS induce the same degree of signaling and natriuresis in the Dahl S and R rats. Although Fedorova and colleagues demonstrated that the increases in circulating CTS are actually greater in the Dahl S than the Dahl R rat,^157,158^ we noted that Dahl S rats had blunted natriuresis as well as minimal evidence for Src activation and Na/K-ATPase and NHE3 redistribution in the proximal tubule, whereas Dahl R rats behaved quite similarly to the wild-type Sprague Dawley rats. We further noted that tubules isolated from the S rats did not respond to ouabain in vitro with Src activation and Na/K-ATPase and NHE3 redistribution, where the proximal tubules isolated from the R rats behaved again quite similarly to those isolated from wild-type Sprague Dawley rats.^18^ We are cautiously optimistic that as we learn more about the biosynthesis and metabolism of cardenolide and bufodienelide CTS, we will ultimately be able to use genetic methods described previously to address their role in salt sensitivity.

## Conclusions

Although the relationship between salt and BP has been known for many years, it is only recently that our understanding of the molecular mechanisms has allowed for potential perturbation of this pathophysiology in experimental animals. It may be hoped that humans share many of these mechanisms with the experimental animals, but further studies must be performed to confirm this hope.
